# A review on multiple sclerosis prognostic findings from imaging, inflammation, and mental health studies

**DOI:** 10.3389/fnhum.2023.1151531

**Published:** 2023-05-11

**Authors:** Jelena Brasanac, Claudia Chien

**Affiliations:** ^1^Charité – Universitätsmedizin Berlin, Klinik für Psychiatrie und Psychotherapie, Berlin, Germany; ^2^Charité – Universitätsmedizin Berlin, Medizinische Klinik m.S. Psychosomatik, Berlin, Germany; ^3^Charité – Universitätsmedizin Berlin, Experimental and Clinical Research Center, Berlin, Germany; ^4^Charité – Universitätsmedizin Berlin, Neuroscience Clinical Research Center, Berlin, Germany

**Keywords:** multiple sclerosis, magnetic resonance imaging (MRI), mental health, depression, neuroinflammation

## Abstract

Magnetic resonance imaging (MRI) of the brain is commonly used to detect where chronic and active lesions are in multiple sclerosis (MS). MRI is also extensively used as a tool to calculate and extrapolate brain health by way of volumetric analysis or advanced imaging techniques. In MS patients, psychiatric symptoms are common comorbidities, with depression being the main one. Even though these symptoms are a major determinant of quality of life in MS, they are often overlooked and undertreated. There has been evidence of bidirectional interactions between the course of MS and comorbid psychiatric symptoms. In order to mitigate disability progression in MS, treating psychiatric comorbidities should be investigated and optimized. New research for the prediction of disease states or phenotypes of disability have advanced, primarily due to new technologies and a better understanding of the aging brain.

## 1. Introduction

**FIGURE 1:**
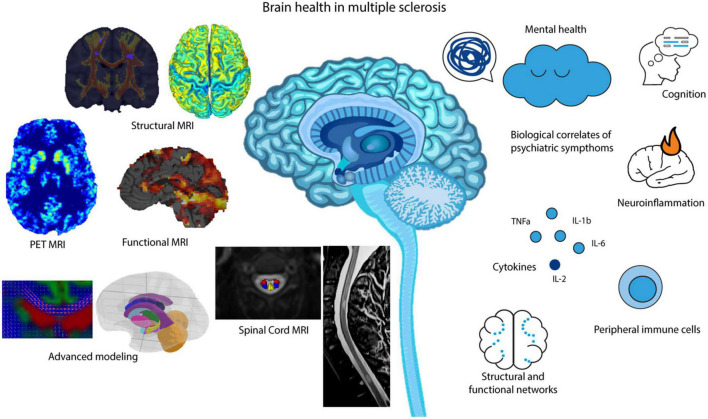
MR imaging, advanced analysis and modeling methods that aid in our understanding of brain health in MS, including cognition, neuroinflammation, structural and functional networks and overall mental health.

Multiple sclerosis (MS) is a neuroinflammatory and chronic disease that affects the brain, spinal cord, and optic nerves (Thompson et al., 2018). With no permanent cure for this neurological disorder, it is a lifelong affection. Patients and clinicians must decide on optimal treatment regimens, which are often changed based on already observable increases in disability (e.g., motor, cognitive, bowel, and bladder) (Gajofatto and Benedetti, 2015; Dalmau, 2018). However, it has long been hypothesized and recently shown in large population-based cohorts, that the earlier this disease is treated, the better the prognostic outcome will be of these patients; even without evidence of disease activity (Stangel et al., 2015; Prosperini et al., 2018; Wandall-Holm et al., 2022). Thus, it would be of interest to all those researching brain health, neurological aging, and predictive modeling in neuroscience to be able to diagnose and treat patients prior to any manifestations of disease-related disability. Moreover, for a better prognostic outcome in MS patients, treatment regimens may also require addressing mental health comorbidities, such as depression. Psychiatric symptoms are common comorbidities in MS and have been shown to interact with the disease course as well as influence patients’ adherence to the therapy (Tarrants et al., 2011; Moore et al., 2012; Marrie et al., 2015; McKay et al., 2018; Binzer et al., 2019). Despite its importance, mental health remains overlooked in this patient group (Marrie et al., 2009). Treating psychiatric symptoms is crucial for developing new treatments for MS, as improving mental health will contribute to the quality of life of MS patients. This mini-review of brain health in MS will aim to give a comprehensive overview of state-of-the-art magnetic resonance imaging (MRI) research that has advanced our understanding of personalized diagnosis and treatment strategies. To further shine a light on this complex neurological condition in relation to brain and mental health, this review will include current research on comorbidities related to MS, such as depression and anxiety (Figure 1).

## 2. Magnetic resonance imaging characteristics

### 2.1. Brain and spinal cord lesions

Magnetic resonance imaging has been used to evaluate lesions in the brain of suspected MS patients for over 2 decades and focused on brain and spinal cord (SC) lesions (Barkhof et al., 1997). In the 2017 revised McDonald Diagnostic Criteria for MS (Thompson et al., 2018), MRI lesion identification in the entire central nervous system (CNS) is a cornerstone for correctly diagnosing patients with a dissemination in time and space. However, due to the complex nature of MS and its unpredictable disease course, lesions in the brain and SC only account for a minor amount of the symptoms observed in patients, termed the “clinico-radiological paradox” (Chard and Trip, 2017). Long-term motor disability is better reflected by location and length of SC lesions (Eden et al., 2019), but lesions can be asymptomatic and longitudinal monitoring with MRI is required (Brownlee et al., 2017; Ciccarelli et al., 2019). Thus, it would seem that lesions, which indicate demyelination in the WM and degeneration of axons in the GM, cannot be the sole imaging marker evaluated when assessing MS patient prognosis (Absinta et al., 2021). Recent advances in MRI technology and analysis methods, disease subtypes, brain and SC gray matter (GM) and white matter (WM) atrophy, WM/myelin analysis, functional MRI networks, along with lesion counts and volumes are able to be evaluated (Sastre-Garriga et al., 2020; Granziera et al., 2021; Romanello et al., 2022).

### 2.2. Brain and spinal cord atrophy

With increased computational research, more accurate parcellations of whole brain/SC GM and WM from MRI can be achieved in MS research (Gros et al., 2019; Guo et al., 2019; Chien et al., 2020). Atrophy in the brain and SC have been shown to relate to normal aging, but also associated with clinical disability in patients with MS when taken together with lesion load (Oh et al., 2014; Opfer et al., 2018). Since the term “atrophy” refers to a decrease in measures of particular regions, it can only be evaluated when comparing two groups or time points. Especially in the last decade, much has been elucidated about which regions of the brain are important to investigate for different MS-related disabilities. Therefore, many recent studies have focused on longitudinal analysis of MRI data to answer questions regarding changes in the brain and SC of MS patients during their disease course.

#### 2.2.1. Brain regions of interest

Previously, MS researchers have focused on using brain atlases to parcellate regions of interest and have found that especially cortical GM and deep GM substructures are shown to be atrophied (Rocca et al., 2010; Narayana et al., 2012; Cooper et al., 2021) leading to more clinical disability accrual. Since whole brain atrophy is also found in normal aging and confounded by sex and head size (Malone et al., 2015; Opfer et al., 2018; Azevedo et al., 2019), it has been proposed to compare MS patients’ predicted “brain age” with that predicted in healthy age- and sex-matched individuals (Raffel et al., 2017). Brain age is commonly calculated by training a machine learning algorithm to create an age regression model using 3D structural MRI (raw or extracted data) as independent values with chronological age as dependent values. Then a multivariable model of healthy brain aging/maturation is constructed which can be applied to new MRI data for “brain age” prediction (Franke and Gaser, 2019). Several studies have found relevance in the brain age gap/difference between the predicted MS brain age and chronological age of the patients, where larger differences in predicted brain age are associated with WM lesion load and more rapid disability progression (Høgestøl et al., 2019; Cole et al., 2020). Recently, it was found that predicted brain age differences also contribute significantly to cognitive performance scores, with larger brain age differences associating with cognitive dysfunction in MS patients (Denissen et al., 2022).

Other studies found that including ventricular cerebrospinal fluid (CSF) volume as an MRI-extracted metric in advanced statistical models increased the prediction of confirmed disability progression (Zivadinov et al., 2016). Also, higher change in lateral ventricular volume over time is associated with disease activity (Millward et al., 2020; Barnett et al., 2021). Recently, the choroid plexus (CP) within the lateral ventricles showed enlarged volumes in MS patients (Gauthier, 2023) and were found to occur in conjunction with chronic lesions and brain atrophy (Klistorner et al., 2022). Enlargement of the CP have also been indicated as markers of inflammatory/acute disease activity (Fleischer et al., 2021; Ricigliano et al., 2021; Margoni et al., 2023); where increased gadolinium enhancement in T1-weighted MRIs (Kim et al., 2020) and T2-weighted intensity have predicted higher disease activity in MS patients (Chien et al., 2022).

#### 2.2.2. Spinal cord regions of interest

It was thought that chronic lesions in the SC lead to atrophy at those specific levels in the cord, since large post-mortem findings of WM affection in the cervical cord was in-line with MS pathology (Gilmore et al., 2005). Although SC lesions and atrophy occur in the cervical cord at the same time (Valsasina et al., 2018), it has recently been shown that there is no such relationship and lesions and whole cord atrophy occur independently from each other in MS (Bussas et al., 2022). Thus, many MRI researchers have begun to evaluate more closely, the location of where atrophy in the cervical cord occurs and found that GM cross-sectional area and the posterior/lateral regions of the WM are most associated with disability as measured by the Expanded Disability Status Scale and different MS subtypes (Schlaeger et al., 2014; Bonacchi et al., 2020; Valsasina et al., 2022). This indicates that perhaps MRI can detect GM and WM regional affection in patients earlier in the disease course than expected, where decreased areas and volumes posteriorly and laterally lead to sensory deficits and motor disability (Mercadante and Tadi, 2022; Natali et al., 2022).

### 2.3. White matter affection

The WM contains the majority of myelin in the CNS and it is well researched that MS is primarily a demyelinating disease (Rosenthal et al., 2020). Axonal loss is evident in smaller fibers of the corticospinal tracts (from the cortex to the lumbar SC regions), and the sensory tracts from the cervical to lumbar SC levels, in post-mortem brain and SC samples (DeLuca et al., 2004). It has been found in every MS stage, many of the WM tracts in the brain are damaged and can be detected using diffusion tensor imaging (DTI) (Preziosa et al., 2011). Fractional anisotropy (FA) can be calculated from DTI-modeled MRI. DTI models the diffusion of water through space, where an FA value closer to 1 indicates restricted water movement (i.e., in axons that are myelinated) and an FA value closer to 0 indicates non-restricted water movement (i.e., demyelinated axons) (Basser and Pierpaoli, 1996). Reduced FA is widespread even in early relapsing-remitting MS and can be observed to decrease over time mostly in the corpus callosum, cerebral peduncle, corticospinal tract, and the posterior thalamic/optic radiation (Asaf et al., 2015). Since WM tracts are connected to GM regions in the brain, it has been of interest to look at how detected WM tract damage can affect the regional GM. One relatively large longitudinal MS study found that over a short period of time (mean follow-up time of 13.7 years), thalamic atrophy could be detected and interestingly, the mean FA in the thalamocortical tracts at baseline could predict the annual thalamic atrophy rate (Weeda et al., 2020). Recently, a longitudinal study in healthy participants observed age-related decreases in WM FA values that are associated with cognitive decline in the dimensions of memory, executive function and general cognition (Coelho et al., 2021). With MS being a demyelinating disease, it could be that the extra WM degradation occurs prior to GM atrophy, which then leads to increased “brain age” estimates and cognitive decline over time.

Diffusion-weighted imaging (DWI) can also be used to model WM tracts using probabilistic tractography, which calculates a connectivity index per voxel in the brain that can be used to evaluate the most likely path of a WM tract with the average number of streamlines that traverse it (Reid et al., 2022). WM lesions, especially those often found in MS, have been thought to impede tractography modeling in MS, thus researchers often use atlas-based versus individual-MRI-based methods (Kuchling et al., 2018). However, it is becoming more common, especially in MS studies, to identify and evaluate which WM tracts are affected by brain lesions using normative high-resolution atlases. These affected WM tracts can be seen as disconnections or as disconnectomes in the brain (Thiebaut de Schotten et al., 2020). These calculated disconnectomes based on individual brain lesion masks have recently been shown to associate well with serum neurofilament light chain levels, a known biomarker of axonal brain damage (Rise et al., 2022).

Another method for evaluating DWI WM tracts is using connectivity matrices calculated between regions of interest (or nodes) in the brain that can indicate less connected nodes using streamline weights and accounting for regional volumes (Kuceyeski et al., 2013). This method includes brain lesion masks that account for structural network modifications (an estimated structural connectivity). One study using this method showed that decreased connectivity in networks structurally related to visual, somatosensory, and attention functions were found in MS patients with higher Expanded Disability Status Scale (EDSS) score (Tozlu et al., 2021). In another study, the modularity, clustering, global and local efficiencies of the WM and GM structural networks were found to differ in a temporal fashion, based on disease duration, indicating that network reorganization occurs along with clinical disability in MS (Fleischer et al., 2017). Probabilistic tractography streamlines have also used to calculate structural connectivity matrices that were used as input features into ensemble machine learning algorithms to predict EDSS score by Barile et al. (2021) Feature importance in predicting disability based on EDSS was also evaluated that highlighted microstructural changes in WM tracts between different cortical regions were related to low, medium, and high clinical disability.

### 2.4. Functional MRI networks

Using resting-state functional MRI (rs-fMRI) and task-based fMRI, researchers have identified “Neural Networks” that are related to different brain functions. By measuring the blood oxygen level dependent (BOLD) signal in the brain using echo planar imaging (EPI) sequences (Kirilina et al., 2016), it is possible to extract information regarding the Amplitude of Low Frequency Fluctuations (ALFF) and regional homogeneity (ReHo) (Lv et al., 2018). These metrics can be used to reconstruct localized regional brain function, where the BOLD signal detected during tasks given to participants indicate specific neuronal activity (Christie et al., 2017). Using this type of functional-connectivity MRI has led to defining a default-mode network, where there is baseline activity detected in rs-fMRI from just wakefulness (Greicius et al., 2004). Recently, it has been found that altered functional brain states and connectivity dynamics, or analysis of the temporal, non-static rs-fMRI networks are related to cognitive decline and clinical disability in MS patients, even at an early stage of the disease (Broeders et al., 2022; Romanello et al., 2022). Task-based fMRI investigations in MS patients have often revealed increased activations of higher-order brain functional areas, such as in classical motor, frontal, and parietal regions, which is thought of as functional networks acting in a compensatory fashion to maintain normal good performance (Rocca et al., 2022). This was recently shown using visual attention paradigms, where higher visual and attention-related, as well as the default-mode network connectivity, was associated with better Brief Visuospatial Memory Test–Revised scores. However, reduced connectivity was found between visual cortical regions with eye-fields (Veréb et al., 2021). Another study hypothesized that task-based fMRI networks may show increased or decreased activation in a disease-related temporal fashion. Indeed, using a visually guided force-matching task, it was found that fMRI activation was lower in the cerebellar, occipital and superior parietal cortical regions, which also correlated with higher EDSS in minimally impaired early MS patients (Strik et al., 2021).

Interestingly, SC lesions have also been found to interrupt the functional connectivity in the cervical cord and may be the pathophysiological reason for disability based on the ventral motor and dorsal sensory networks in later MS (Conrad et al., 2018). These findings suggest there will become a larger role for rs- and task-based fMRI in evaluating brain and CNS health of MS patients in the future (Nejad-Davarani et al., 2016).

## 3. Mental health and MS

In MS patients, psychiatric symptoms are common comorbidities, with depression being the main one (Marrie et al., 2015; Gold et al., 2020). In addition to withholding pharmacological treatment and rehabilitation programs, depressive symptoms result in worsening functional outcomes (Binzer et al., 2019). Even though these symptoms are a major determinant of quality of life in MS, they are often overlooked and undertreated (Marrie et al., 2009). MS patients have a 30.5% prevalence for developing depression and 22.1% for anxiety, with clinically significant depressive or anxiety-related symptoms found in 35% and 34% of patients (Boeschoten et al., 2017). Prevalence for depression raises up to 44.5% during relapses and lifetime prevalence increases to 50% (Feinstein, 2011). Clinically isolated syndrome (CIS) and early MS patients from a meta-analysis of 51 studies were reported to exhibit depressive and anxiety-based symptoms in 17% and 35% of patients, respectively (Rintala et al., 2019).

There has been evidence of bidirectional interactions between the course of MS and comorbid psychiatric symptoms. Two large longitudinal cohort studies have suggested that the presence of psychiatric comorbidities increased the risk of MS disability progression (McKay et al., 2018; Binzer et al., 2019). In the retrospective cohort study from the Canadian provinces of British Columbia and Nova Scotia, McKay et al. (2018) followed 2,312 incident cases of adult-onset MS for around 10 years and found 38.5% of participants met the criteria for mood and anxiety disorders. They were associated with subsequent neurologic disability progression as measured by the EDSS score and the effect was statistically significant among women but not men. Interestingly, more than 40% of these MS patients met the criteria for comorbid psychiatric disorder prior to the MS onset. With 37%, the most prevalent psychiatric comorbidity was depression, while anxiety was present in 22.1% and bipolar disorder in 5.1% of MS patients. A further separate analysis of the individual effect of each psychiatric comorbidity showed that only depression was significantly associated with higher EDSS scores. The results of a Swedish cohort study by Binzer et al. (2019) showed that MS patients with depression, defined as being diagnosed with depression or requiring treatment with antidepressants, have faster disease progression than non-depressed MS patients. Moreover, they reported significant results for both men and women, indicating that the lack of significant results for men in the Canadian study could have been because of the small number of male participants. A nested case-control study with 10,204 incident MS cases investigated the occurrence of different symptoms in MS versus healthy participants from the first record of the disease and up to 10 years before it (Disanto et al., 2018). MS patients showed a significantly higher risk of being diagnosed with depression up to 10 and with anxiety up to 5 years before the disease onset. Disanto et al. (2018) found that psychiatric symptoms were reported years before the first record of the disease, which suggests mood disturbances are not a consequence of the pathology, but rather an integral part of it (Feinstein et al., 2014). On the other hand, clinical relapses and neurological disability in MS have been linked with higher rates of depressive symptoms (Moore et al., 2012). In a study by Moore et al. (2012) that looked at clinically significant depression symptoms during and post-MS relapse the point prevalence of depression during a confirmed MS relapse was 44.5%. It significantly decreased but stayed high in the follow-up, 29.2% at two and 34.4% at 6 months follow-up, suggesting that although the improvement in disability leads to improvement of depressive symptoms, they still stay persistent and high.

### 3.1. Biological correlates of psychiatric symptoms in MS

In order to mitigate disability progression in MS, treating psychiatric comorbidities should be investigated and optimized. Studies investigating biomarkers of psychiatric symptoms in MS may be relevant for understanding the biology that underlies the comorbidity of inflammatory and mood disorders with implications for prevention and treatment. Neuroinflammation seems to be one of the major correlates as studies show that hippocampal neuroinflammation, measured as microglial activation in the hippocampus (Colasanti et al., 2016) was related to depression in MS patients. Moreover, the data show how subclinical intrathecal inflammation, even without detrimental symptoms, can induce mood alterations (Rossi et al., 2017), which can further predict inflammatory reactivation in MS relapses. Relapsing MS patients showed greater values for state anxiety and depression [measured as State-Trait Anxiety Inventory (STAI)-state and Beck’s Depression Inventory II (BDI II)] in comparison to the remitting MS patients, but similar trait anxiety scores. Along with the reduction of neuroinflammation, Rossi et al. (2017) found that there was a reduction in state anxiety and depression scores suggesting that (subclinical) inflammation affects anxiety and depression in MS. Taken together, this suggests that inflammation may be a critical biological event involved in mood disorders and in MS. When it comes to studies investigating proinflammatory cytokines, known to be associated with depression severity, there is evidence for the correlation of cytokines in cerebrospinal fluid (CSF) and mood changes. The levels of tumor necrosis factor-alpha (TNF-a) interleukin-1 beta (IL-1b) (Rossi et al., 2017), and interleukin 6 (IL-6) (Brenner et al., 2018) were found to be associated with depression severity as measured by BDI II, while levels of Interleukin-2 (IL-2) were found to be correlated with anxiety measured by STAI-state. A study investigating cellular frequencies in the peripheral immune system identified CD4+ T central memory cells expressing low levels of CCR7+ as a robust biological correlate of MS-associated depression. These cell frequencies were correlated with depression severity, but not MS disease severity, and associated with neuroinflammation measured as lesion load on MRI (Brasanac et al., 2022). Thus, indicating inflammation and immune system changes as biological pathways implicated in the shared pathobiology of MS and depression (mood and immune disorders). Among other biological correlates of MS-associated depression, studies have pointed to hypothalamic-pituitary-adrenal axis hyperactivity (Gold et al., 2011), hippocampal atrophy (Gold et al., 2014), and larger cerebral T2 lesion load (Feinstein et al., 2004). Furthermore, a study testing the association of depression and lesions in amygdala-prefrontal fiber tracts found that depressed MS patients were less able to regulate negative emotions, indicating that emotional regulation in MS-associated depression was affected by lesion load (Meyer-Arndt et al., 2022). In addition, disruption in frontal–parietal white matter tract over the course of 5 years could predict a diagnosis of depression in multiple sclerosis (Ashton et al., 2021).

### 3.2. Cognition and age in MS-related depression

Studies have shown that severely depressed MS patients have difficulties with working memory (Arnett et al., 1999a,b), information processing speed (Lubrini et al., 2012), and executive functioning (Arnett et al., 2001; Feinstein et al., 2014). The biology underlying this comorbidity could be the atrophy of the hippocampus as one study has shown that enlarged temporal horns are linked to depression and consolidation deficits in memory tasks (Kiy et al., 2011). In a large cohort of MS patients with 13,821 individuals, depressive symptoms were found to be correlated with worse cognitive performance measured as information processing speed, manual dexterity and walking speed (Chan et al., 2021). This association was related to the age of participants, younger MS patients experiencing moderate to severe MS showed slower processing speed while older MS patients had slower walking speed. In the animal model of MS, the experimental autoimmune encephalomyelitis (EAE), data show that changes in cognition and behavior appear even before demyelination starts and correlates with cytokines TNFa and IL1b in the hypothalamus, as well as with corticosteroid hormone levels (Acharjee et al., 2013).

When it comes to the link between psychiatric symptoms and age in MS, results are ambiguous. Several studies are showing a trend toward a decreasing prevalence of depressive symptoms with increase in age of MS patients (Patten et al., 2003; Garcia and Finlayson, 2005). Furthermore, data show that younger age at onset is correlated with the occurrence of depression in MS patients (Beiske et al., 2008) and that the shorter the duration of MS (relapses) the higher the risk for depression (Williams et al., 2005). A possible explanation for the change in prevalence of depression could be the underlying biology of MS. While depression has been associated with inflammation in the early phases of the disease characterized by relapses, with age inflammatory processes are replaced with neurodegeneration characterized by motor and cognitive disability (Gold and Irwin, 2009; Musella et al., 2018). In contrast with these results are studies reporting a positive association between depression and age of MS patients (da Silva et al., 2011; Mattioli et al., 2011), while several other studies did not find any relationship between age and depression in MS. More studies are needed to further investigate and elucidate this aspect of MS.

## 4. Discussion and future directions

Increasingly more research is going into the neuroinflammatory and metabolic substrates involved in MS, using positron emission tomography (PET) imaging. PET imaging gives information about metabolism of *in vivo* organs, such as the brain, since it uses radiotracers that are actively taken up into tissues in active catabolism or can bind to receptors on cell surfaces (Politis and Piccini, 2012). PET tracer detection was initially conducted using computed tomography (CT) machines due to its function in detecting X-ray photons (radiation); however, hybrid PET/MRI machines are becoming more prominent in the research field due to the superior contrast that MRI can image between soft-tissues (Antoch and Bockisch, 2009). Since PET allows for identification of active metabolism, researchers have begun to evaluate its capability in visualizing task-based and resting-state networks (Rischka et al., 2018; Fang et al., 2021). Very recently, an amyloid radiotracer (11C-PiB) (Rabinovici et al., 2007), which also has a high capacity to bind to myelin, was used to longitudinally evaluate remyelination in MS patient brains (Tonietto et al., 2023). Tonietto et al. (2023) found that enlargement of the CP was associated with failure to remyelinate periventricular WM leading to possible regional cortical atrophy in relapsing-remitting MS patients.

With new technological advancements, machine learning (ML) and deep learning in CNS disorders have become more popular, especially using MRI data due to its complex extraction of metrics and 3D or 4D nature (4th dimension is related to the temporal aspect as in fMRI). There have been several studies in the last few years that have given rise to interesting information related to brain health and MS. Recently, it has been found that ML can be used to predict MS future disease activity in patients using unprocessed MRIs, where within the periventricular region and CP there seems to be higher T2-weighted intensities in people with higher future disease activity (Chien et al., 2022). Along the same line, unsupervised ML in combination with advanced statistical methods have been used to identify different MS-related MRI-extracted subtypes that lead to different confirmed disability progression and relapse rates (Eshaghi et al., 2021). Also, using a deep learning algorithm trained on unprocessed Alzheimer’s brain MRIs, one study found a way to transfer this learned algorithm (transfer learning) to identify MS brains versus healthy participant brains (Eitel et al., 2019). Thus, it can be seen that the assessment of brain health and patient prognosis in MS is moving toward the use of more advanced imaging related to networks and use of ML and deep learning technologies.

Even though the majority of MS patients have comorbid mental health disorders there are not so many studies exploring their long-term effects (McKay et al., 2018). Having a non-treated comorbid psychiatric disorder may hamper adherence to disease-modifying therapies (Tarrants et al., 2011) or promote unhealthy coping strategies (McKay et al., 2016) in MS patients. It is of paramount importance to treat comorbid mood disorders in MS as mental health represents one of the major determinants of the quality of life (Marrie et al., 2009) and underlying mood disorder is associated with suicidal ideation in this population at a rate higher than the general population (Feinstein and Pavisian, 2017). For developing new treatments for MS patients and for successful therapy, it is crucial to understand and treat psychiatric symptoms as well. Therefore, the way forward could be to combine therapies targeted for a specific disease phenotype, such as MS-associated depression.

Although depression has been identified as one of the main mental health disorders found in MS, high comorbidity with mental health disorders is not specific to MS or limited to inflammatory diseases. Besides MS, other chronic conditions such as cardiovascular, neurological, and metabolic disorders also share depression as one of the most frequent accompanying comorbidities (Gold et al., 2020). Contributing factors to this comorbidity range from shared genetics to converging underlying biology, as well as various psychological and lifestyle factors. Numerous studies in recent decades have compounded evidence that points to the contributions of the immune system and inflammation in developing mood disorders (Beurel et al., 2020). Recently, more emphasis on identifying subtypes of depression has been made in mental health research. For example, the subtype of inflammatory depression has been identified (Lynall et al., 2020), which also involves depressed patients with underlying inflammatory disorders such as MS. Further investigation of these disease subtypes can help identify patient populations that can benefit from targeted therapeutic strategies.

In the last decades, imaging studies in the field of depression research have significantly grown in number (Zhuo et al., 2019). However, real progress has been hampered by underpowered studies and a lack of reproducibility. To address these issues ENIMGA MDD consortium was formed a decade ago and they have been able to identify subtle structural brain changes related to distinct clinical and demographic characteristics of depression (Schmaal et al., 2020). Despite new technological advancements in MRI and mental health research in MS, there are still limitations particularly in answering questions about psychiatric disorders. In almost all MRI studies, there are confounding variables that are required to be considered, such as age (Opfer et al., 2018), sex (Voskuhl et al., 2020; Chyzhyk et al., 2022), and often brain lesion load (Sinnecker et al., 2012). However, with more and more confounders, the importance of variables of interest in predicting outcomes may be lost or become biased, especially in machine learning applications (Snoek et al., 2019). Larger and standardized benchmark MRI datasets may help reduce bias and increase prediction accuracies (Eitel et al., 2021). There is also speculation that our general lack of understanding of the mechanisms of psychiatric illnesses is a potential reason as to why we have no concrete neurobiological insights from over 30 years of functional neuroimaging (Nour et al., 2022). Thus, it will be important to move toward a more holistic understanding of neurobiology and mental health by conscientiously designing prediction models that inherently reduce bias and allow for comprehensive real-world data usage (Hooker, 2021).

## Author contributions

JB and CC contributed to the writing of the original draft, read, and approved the submitted version.
